# Cognitive Empathy Differences on the Questionnaire of Cognitive and Affective Empathy Distinguish Between Borderline Personality Disorder and Other Mental Disorders

**DOI:** 10.1192/bjo.2025.10196

**Published:** 2025-06-20

**Authors:** Ariana Axiaq, Donald MacIntyre, Douglas Steele, David Hayward

**Affiliations:** ^1^University of Edinburgh, Edinburgh, United Kingdom; ^2^University of Dundee, Dundee, United Kingdom; ^3^St John’s Hospital, Livingston, United Kingdom

## Abstract

**Aims:** Bipolar personality disorder (BPD) is associated with a deficiency in cognitive empathy, defined as the ability to infer other’s mental state by imagining their perspective and interpreting cues like facial expression. However, patients with BPD tend to have a typical or heightened emotional empathy – having reciprocal feeling state. We hypothesise that an empathy measure that discriminates between cognitive and affective empathy could aid diagnosis, quantify severity, inform prognosis, and stratify treatment of BPD.

The Questionnaire of Cognitive and Affective Empathy (QCAE) was produced by assimilating the most discriminating aspects of other well-validated questionnaires. It clearly defines empathy and is easy to use. The QCAE has also been shown to capture the characteristic empathy difference in people with BPD compared with non-clinical controls, but studies using non-clinical controls cannot determine whether these empathy differences discriminate between different mental disorders or are generally symptomatic of mental distress. Therefore, we measured empathetic aptitude using the QCAE in a BPD group and comparable group of people with other mental disorders.

This study aims to assess whether empathetic amplitude – cognitive and emotional empathy scores on QCAE, is different in BPD compared with other mental health disorders.

**Methods:** Participants diagnosed with BPD were recruited in outpatient appointments and in inpatient settings. Diagnoses were affirmed using DSM–IV diagnostic criteria by consultant psychiatrists. QCAE results were compared with a clinical control group with other mental disorders, also recruited in outpatient and inpatient settings.

**Results:** In the BPD group: N=40 (38 female), cognitive empathy mean on QCAE was 35.075 (SD 7.917) whereas emotional empathy mean was 46.80 (SD 12.90). Meanwhile in the clinical control group: N=23 (9 female, depression 5, schizophrenia 10, dissociative disorder 1, mania 4, NDD 2, delusional disorder 1), cognitive empathy mean was 55 (SD 10.531) while emotional empathy averaged at 35.609 (SD 6.103). There was a significant cognitive empathy score difference between the control and BPD group (*p=0*.012), with Cohen’s d of 0.696, the difference in emotional empathy was not significant (*p=0*.781).

**Conclusion:** These results corroborate the characteristic BPD empathy difference of an impaired cognitive empathy but a typical emotional empathy; people with BPD struggle to understand the motives and intentions of others, but their own emotions can be roused. This convincingly explains why it can be difficult for affected people to navigate interpersonal challenges. QCAE empathy testing could add objectivity to the otherwise subjective BPD diagnosis. However, prospective trials are needed to determine the prognostic utility of the QCAE tool.

